# Pulsed-field Gel Electrophoresis for *Salmonella* Infection Surveillance, Texas, USA, 2007

**DOI:** 10.3201/edi1606.091276

**Published:** 2010-06

**Authors:** Stephen G. Long, Herbert L. DuPont, Linda Gaul, Raouf R. Arafat, Beatrice J. Selwyn, Joan Rogers, Eric Casey

**Affiliations:** Houston Department of Health and Human Services, Houston, Texas, USA (S.G. Long, R.R. Arafat, J. Rogers); University of Texas School of Public Health, Houston (H.L. DuPont, B.J. Selwyn); St. Luke’s Episcopal Hospital, Houston (H.L. DuPont); Baylor College of Medicine, Houston (H.L. DuPont); Texas Department of State Health Services, Austin, Texas, USA (L. Gaul, E. Casey)

**Keywords:** Salmonella, bacteria, pulsed-field gel electrophoresis, PulseNet, surveillance, epidemiology, Texas, enteric infections, microbiology, dispatch

## Abstract

To identify sources of transmission for area clusters, in 2007 the Houston Department of Health and Human Services conducted an 8-month study of enhanced surveillance of *Salmonella* infection. Protocol included patient interviews and linking the results of interviews to clusters of pulsed-field gel electrophoresis patterns detected by the local PulseNet laboratory.

To detect *Salmonella* clusters, public health laboratories perform pulsed-field gel electrophoresis (PFGE) that provides a PFGE pattern, or DNA fingerprint. If the PFGE patterns of isolates from >2 persons are indistinguishable, the responsible bacteria may be related to a common source (*1**–**3*)*.* PulseNet is a network of public health laboratories coordinated by the Centers for Disease Control and Prevention (CDC), in which bacteria that cause foodborne diseases, including *Salmonella* isolates, are analyzed by using PFGE. This network provides the means to rapidly compare PFGE patterns from isolates submitted in different geographic areas. State and local laboratories upload PFGE patterns to the national CDC PulseNet database. Indistinguishable patterns at the national level might represent a large multistate outbreak (*4*–*6*).

As a city health department located in the state of Texas, the Houston Department of Health and Human Services (HDHHS) investigates all local *Salmonella* cases to detect outbreaks and vehicles of transmission. The HDHHS laboratory has been certified as a PulseNet laboratory since 2001 and serves residents of Houston (≈2.1 million persons) and adjacent counties.

Because PFGE patterns obtained by a local health department may appear to be sporadic or unrelated to a more generalized process (*2*), local public health practitioners may gain a larger perspective by receiving notification of state and national clusters (*4*,*5*). During 2002–2005, before this study was conducted but during a time HDHHS was in routine communication with PulseNet, most local PFGE patterns were not recognized as linked to statewide or nationwide clusters.

In this study, HDHHS sought to determine more rigorously the utility of PFGE in local surveillance (as opposed to national surveillance) in detecting area clusters and vehicles of transmission. Another goal was to determine how local PFGE patterns and clusters are associated with larger-scale clusters. The study was approved by the Committee for the Protection of Human Subjects, University of Texas Health Science Center.

## The Study

During an 8-month period, May 1 through December 31, 2007, HDHHS received 145 *Salmonella* case reports in which patients resided in Houston. The HDHHS laboratory performed PFGE for 106 (73%) isolates from the Houston case-patients. The laboratory performed PFGE for all isolates it received. The remaining 39 Houston cases had been reported by providers that did not forward the isolate to HDHHS. The HDHHS laboratory used a standardized PulseNet *Salmonella* protocol for PFGE and compared PFGE patterns for these isolates by using Bionumerics 4.0 software (Applied Maths, Sint-Martens-Latem, Belgium). Using a hypothesis-generating questionnaire, immediately upon receiving the case the first author interviewed 96 (91%) of the 106 case-patients with an assigned PFGE pattern. Follow-up was not feasible for the remaining 10 case-patients. Table 1 provides the demographic characteristics of the 106 case-patients. The HDHHS laboratory posted the PFGE patterns weekly to HDHHS epidemiologists, who then further investigated the clusters attempting to identify common sources.

**Table 1:** Cases of *Salmonella* infection reported to HDHHS, incidence rates, and PFGE results, May 1, 2007–December 31, 2007*

Case-patient characteristic	No. cases reported to HDHHS (%), n = 145	Incidence rate,† n = 145	No. (%) case-patients assigned a PFGE pattern, n = 106	No. (%) case-patients assigned a PFGE pattern and interviewed, n = 96
Sex				
M	65 (44.8)	10.0	50 (47.2)	45 (46.9)
F	80 (55.2)	12.3	56 (52.8)	51 (53.1)
Age, y				
<1	27 (18.6)	119.3	21 (19.8)	19 (19.8)
1–4	40 (27.6)	47.3	31 (29.2)	27 (28.1)
5–19	21 (14.5)	7.3	11 (10.4)	11 (11.5)
20–34	9 (6.2)	2.6	7 (6.6)	7 (7.3)
35–54	20 (13.8)	5.5	15 (14.2)	13 (13.5)
55–74	16 (11.0)	10.4	13 (12.3)	11 (11.5)
>75	12 (8.3)	25.4	8 (7.5)	8 (8.3)
Race/ethnicity				
White				
Non-Hispanic	37 (25.5)	9.2	27 (25.5)	25 (26.0)
Hispanic	70 (48.3)	14.4	45 (42.4)	43 (44.8)
Black	23 (15.9)	7.1	20 (18.9)	17 (17.7)
Asian	9 (6.2)	13.1	9 (8.5)	9 (9.4)
Unknown	6 (4.1)	‡	5 (4.7)	2 (2.1)
Total	145	11.1	106	96

*HDHHS, Houston Department of Health and Human Services; PFGE, pulsed-field gel electrophoresis. †Rate was calculated as number of cases/100,000 population/year, based on the 8-month study period. ‡Rate was not calculable.

Epidemiologists considered a group of *Salmonella* cases to be a cluster if 1) PFGE patterns of all isolates were indistinguishable; and 2) specimens were collected each within 90 days of at least 1 other case. A more inclusive 90-day interval was used, rather than the 60-day interval used by PulseNet, because the number of cases in a local PFGE cluster is typically small. A case that was not in a cluster was considered a singlet case.

Analysis of 106 *Salmonella* isolates from Houston residents yielded 74 distinctive PFGE patterns, of which 66 were forwarded to the Texas Department of State Health Services (DSHS) for comparison with the DSHS laboratory’s database and to further identify clusters. Eight singlet patterns were not further analyzed because of lack of staff in the laboratories. The DSHS returned a list of state ID numbers and county of residence for case-patients with matching isolate PFGE patterns, and HDHHS and DSHS epidemiologists conferred about the data.

Of the 106 *Salmonella* cases with identified PFGE patterns, 42 assembled into 10 clusters, with 2–13 cases per cluster. PFGE patterns for 8 of these clusters matched patterns in the DSHS statewide database, and patterns of 5 clusters matched those in other states obtained during the same period (Table 2).

**Table 2:** Ten *Salmonella* pulsed-field gel electrophoresis clusters among residents of Houston, Texas, USA, and 2 Houston singlet cases linked by PFGE to national outbreaks, detected May 1, 2007–December 31, 2007*

Serotype	PFGE pattern, *Xba*l†	No. cases in Houston	No. other cases in DSHS database	Associated national outbreak	Common exposure or other link
Braenderup	JBPX01.0516	2	4	PulseNet outbreak 0708HUJBP-1c	Traveled or resided in southern Texas
Corvallis	SCVX01.0014	2	0	–	Unknown
Enteritidis	JEGX01.0004	13	Numerous	PulseNet outbreak 0801PAJEG-1	Egg consumption
Enteritidis	JEGX01.0005	6	25	–	Unknown
Infantis	JFXX01.0022	3	5	–	Unknown
Infantis	JFXX01.0041	5	1	–	Unknown
Paratyphi b var. java	JKXX01.0014	4	2	PulseNet outbreak 0710NCJKX-1c (*7*)	Contact with miniature turtles
Typhimurium	JPXX01.0276	2	0	–	Unknown
Typhimurium	JPXX01.0621	3	3	PulseNet outbreak 0801ORJPX-1c	Unknown
Typhimurium	JPXX01.0006	2	3	Possible bovine outbreak (multistate) 0708MLJPX-1c	Unknown
Typhimurium	JPXX01.1037	1	0	PulseNet outbreak 0704WIWWS-1c	Packaged vegetable product‡
Typhimurium	JPXX01.1354	1	1	PulseNet outbreak 0703MLJPX-2c	Contact with hamsters‡

*PFGE, pulsed-field gel electrophoresis; DSHS, Texas Department of State Health Services. †PulseNet nomenclature. ‡The case was linked by PFGE to a PulseNet cluster, but the patient denied having been exposed to the hypothesized epidemiologic link.

HDHHS identified a likely exposure for 3 local PFGE clusters (Table 2). The first cluster, *S. enterica* serovar Braenderup JBPX01.0516 (PulseNet nomenclature), included 2 Houston case-patients and 5 case-patients residing in adjoining counties. Two persons reported travel to Matamoros, Mexico, before getting sick. Four isolates in the DSHS database had this PFGE pattern, of which 3 had been obtained from case-patients who resided in Brownsville, Texas, near Matamoros. HDHHS posted the PFGE pattern on PulseNet Listserve, and the Ohio Department of Health responded with information regarding a concurrent outbreak of the same strain in a church group whose members became ill while visiting southern Texas. *S. enterica* serovar Enteritidis JEGX01.0004, one of the most common patterns in the HDHHS and DSHS PFGE databases, was noted by HDHHS to be occurring at above expected levels in December 2007. The Pennsylvania Department of Health posted outbreak clusters in Pennsylvania with the same strain, associated with the consumption of improperly cooked eggs. Nine of the 13 (69%) Houston case-patients reported eating eggs during the week before illness onset. In 2 Houston households, persons became sick after eating eggs purchased in farmers’ markets. The North Carolina Division of Public Health linked a third PFGE cluster, *S*. *enterica* serovar Paratyphi B var. Java, JKXX01.0014, to miniature turtles (*7*)*.* Two of 4 Houston patients and another patient in Victoria, Texas, reported having contact with miniature turtles.

During the 8-month study, the HDHHS laboratory also sent patterns for 56 (87%) of the 64 singlet isolates to DSHS, which coupled 11 (20%) of these with more cases in their statewide database. Isolates from 2 Houston singlet cases had patterns matching 2 concurrent multistate outbreak patterns. An isolate of *S. enterica* serovar Typhimurium JPXX01.1037 matched a PulseNet PFGE cluster pattern attributed to a nationally distributed packaged vegetable product. The other isolate, *S. enterica* serovar Typhimurium JPXX01.1354, matched a pattern linked to an outbreak investigated by Wisconsin Division of Public Health in which case-patients were exposed to hamsters. For these singlets, HDHHS was unable to confirm an epidemiologic link between the Houston case and the national outbreak (Table 2).

## Conclusions

Using PFGE patterns, HDHHS discerned vehicles of transmission for local clusters. Such findings could enable a local health department to intervene to address outbreaks currently in progress. Even small clusters are strong indicators because the actual number of cases in an outbreak is typically vastly larger.

Consistent cooperation between HDHHS and DSHS epidemiologists enabled them to see Houston PFGE patterns in a context of statewide and national patterns and clusters. A Houston PFGE pattern that was part of a local cluster was quite likely to match a DSHS (statewide) or CDC (national) pattern. This finding is in contrast to results for 56 singlet patterns; only 11 were found to match patterns of cases outside the local area.

Analysis of PFGE clustering assisted this surveillance system in detecting outbreaks successfully. Findings on PulseNet helped HDHHS epidemiologists identify sources of bacteria in local clusters. HDHHS conducted prompt interviews of 91% of the Houston patients. Of course, a 100% follow-up would have been better, but this study demonstrates the successes that are possible through routine surveillance by a local health department, given its resources. In an ideal situation, a PulseNet-certified laboratory performs local surveillance in sustained close cooperation with epidemiologists who conduct timely investigations based on laboratory findings.
